# Advancing first-contact, primary care physiotherapist practice: the value of integrating point-of-care diagnostic tools

**DOI:** 10.3389/fresc.2025.1704822

**Published:** 2025-12-08

**Authors:** Nathan J. Savage, Richard P. Nielsen, Art Nitz, Mohini Rawat, Bruno U. K. Steiner

**Affiliations:** 1 Department of Physical Therapy, Winston-Salem State University, Winston-Salem, NC, United States; 2 Rocky Mountain University of Health Professions, Provo, UT, United States; 3 Noorda College of Osteopathic Medicine, Provo, UT, United States; 4 Rocky Mountain University of College of Optometry, Provo, UT, United States; 5 Physical Therapy Division, University of Kentucky, Lexington, KY, United States; 6 American Academy of Musculoskeletal Ultrasound, New York, NY, United States; 7 Washington Center for Bleeding Disorders, Washington Institute for Coagulation, Seattle, WA, United States; 8 Department of Rehabilitation Medicine, University of Washington, Seattle, WA, United States

**Keywords:** electrodiagnostic testing, first-contact, point-of-care diagnostic testing, primary care, ultrasound imaging, advanced practice physiotherapy, direct access, diagnostic precision

## Abstract

Healthcare systems worldwide face mounting challenges, including shortages of primary and specialty care providers, escalating direct and indirect medical costs, and limited access to high-quality diagnostic and interventional services—burdens that disproportionately affect patients in rural or medically underserved areas. Because timely, reliable, and affordable diagnostic and interventional services are essential to comprehensive, lifelong care, the expanding role of physiotherapists as autonomous, first-contact providers represents a critical and timely innovation. This article underscores the value of electrodiagnostic (EDX) testing and ultrasound imaging (USI) as instrumented point-of-care diagnostic tools available to physiotherapists through recognized pathways of training and credentialing and argues for their greater integration into contemporary practice. The authors contend that expanding training opportunities in both entry-level physiotherapy curricula and post-graduate programs will increase physiotherapist utilization of these technologies, strengthen the evidence-based rationale for physiotherapy as a primary care profession, and solidify the discipline's role as an indispensable member of the interdisciplinary healthcare team. By broadening access to EDX testing and USI, physiotherapists can deliver timely and cost-effective diagnostic and interventional services, mitigate barriers created by provider shortages, and help modernize healthcare systems to better meet the demands of strained populations worldwide.

## 1 Introduction

The American Physical Therapy Association's *Guide to Physical Therapist Practice* (APTA's Guide) states that “Physical therapists are health professionals who diagnose and manage movement dysfunction as it relates to the restoration, maintenance, and promotion of optimal physical function and the health and well-being of individuals, families, and communities.” (1) Physiotherapists have long been recognized as medical professionals addressing the health and wellness needs of patients and clients across the lifespan (2, 3). As experts in movement-related dysfunction, physiotherapists examine and evaluate impairments involving the neuromusculoskeletal system and the associated functional deficits that negatively impact an individual's quality of life (1).

Physiotherapists utilize a variety of tests and measures when examining patients, including joint goniometry and inclinometry, manual muscle testing and force dynamometry, reflex and sensory testing, balance and gait analysis, passive joint mobility testing, and symptom provocation tests (4, 5). Evaluating the results of these tests assists in the establishment of a physiotherapy diagnosis/classification and the associated prognoses, informs the selection of appropriate manual therapy and therapeutic exercise interventions and modalities, and guides clinical decision-making in a patient's medical management including consultation, co-management, and outside referral.

The APTA's Guide provides evidence-based descriptions of what contemporary physiotherapist practice *can and should entail* and specifically identifies electrodiagnostic (EDX) testing and ultrasound imaging (USI) as instrumented tests used to assess nerve integrity, motor and muscle function, joint and skeletal integrity, posture, circulation, and soft tissue injury, among others.

These instrumented point-of-care diagnostic tools are a logical extension of the physiotherapy examination and evaluation, enhancing diagnostic precision and confirming clinical impressions (6–18). EDX testing and USI benefit patients by enabling timely evaluation of neuromusculoskeletal complaints and reducing burdensome visits to outside providers (19–28).

Recently, the APTA published a report highlighting the economic value of physiotherapy in the United States (U.S.) that applies to physiotherapist practice internationally demonstrating that physiotherapist services provide cost-effective care that improves health and quality of life, consistent with the profession's vision of “transforming society by optimizing movement to improve the human experience” (2, 29).

The global healthcare landscape is burdened with primary and specialty care provider shortages, high direct and indirect medical costs, and limited access to quality diagnostic and interventional services, a problem that is particularly keen for patients that live in rural or medically underserved areas (21, 23, 24, 30, 31). Access to reliable and affordable diagnostic and interventional services is necessary for patients to receive comprehensive healthcare across their lifespan, making the expanding role of physiotherapists as autonomous first-contact, primary care providers a welcome and needed innovation in the health, wellness, and medical sectors (21, 23, 24, 26, 32–34).

World Physiotherapy, the global body representing national physiotherapy associations, asserts that physiotherapists are educated and competent to serve as first-contact practitioners capable of autonomous evaluation, diagnosis, intervention, and discharge without the need for medical referral Physiotherapists providing first-contact services improve access to care, reducing geographic, time, and financial barriers for patients while delivering cost-effective management across healthcare systems (21, 23, 24, 26, 31–35).

Integration of EDX testing and USI allows physiotherapists to enhance diagnostic precision, improve patient access to timely and appropriate interventions, and strengthen the value of first-contact care in strained healthcare systems (14, 23, 31).

Among the guiding principles of the physiotherapy profession's vision are *Collaboration* and *Innovation* (36). Physiotherapists should coordinate care across disciplines and provide timely referral when necessary (20–22, 24–26, 37).

Patients in the U.S. have direct access to physiotherapist services in all 50 states, District of Columbia, and U.S. Virgin Islands with no restrictions to patient access in 21 states, providing the infrastructure for expanded first-contact care (38).

First-contact, primary care physiotherapist practice, pioneered in the U.S. military in the 1970s and implemented in civilian and military systems, has been shown to be safe, effective, improve patient satisfaction, reduce healthcare utilization, and support timely, cost-effective triage and management (19, 21–23, 25–28, 32, 37, 39–42)

These outcomes align with the *APTA's Strategic Plan 2022-2025* and *World Physiotherapy's* vision, promoting first-contact access and self-referral to physiotherapy services across healthcare systems (35, 36).

The authors agree with the APTA House of Delegates recently updated language regarding physiotherapist services in first-contact, primary care settings, which states that “Physical therapists possess clinical expertise in both disease prevention and management of health to evaluate and manage common conditions seen in primary care settings. Physical therapists practicing to the full extent of their education and clinical training in primary care improve the health needs of society” (HOD P06-18-28-22). Furthermore, the authors recognize that to accomplish the vision of the physiotherapy profession and gain widespread acceptance as first-contact, primary care providers that skilled use and interpretation of advanced diagnostic tools is likely a necessity.

The purpose of this article is to highlight the value of EDX testing and USI as instrumented point-of-care diagnostic tools with recognized paths for training and credentialing including board certification [Minimum Eligibility Requirements and General Information for All Physical Therapist Specialist Certification, specialization.apta.org; Musculoskeletal Ultrasound (MSK) Examination for Physicians, http://www.apca.org].

As board-certified specialists with 143 + years of collective experience using EDX testing and USI, the authors argue that contemporary physiotherapist practice requires integrating these tools into entry-level education and providing robust post-graduate training opportunities.

Expanded opportunities for training in EDX testing and USI will enhance clinical utilization, further establish physiotherapy as a first-contact, primary care profession, and improve patient access to timely, cost-effective diagnostic and interventional services in increasingly strained healthcare systems.

## 2 Instrumented diagnostics in physical therapist practice: a history

### 2.1 Electrodiagnostic testing

Point-of-care EDX testing, consisting of peripheral nerve conduction studies and needle electromyography (jointly “electroneuromyography”), evaluates the integrity of the neuromuscular system. Conducted as an extension of the clinical examination and not as a laboratory procedure, EDX testing is considered by many a primary diagnostic procedure for objectively assessing nerve and muscle integrity and identifying pathology relevant to neuromuscular differential diagnosis (43–47).

Unlike most imaging modalities that produce static images of soft tissue structures for later review by another provider, EDX testing evaluates neuromuscular *function* in real-time through the measurement of peripheral sensory and motor nerve responses (e.g., distal latency, conduction velocity, and amplitude) and the electrical activity in skeletal muscle at rest and during volitional activation (e.g., patterns of motor unit recruitment and motor unit morphology) (10, 13, 43, 45, 46).

EDX testing demonstrates high reliability and validity, extending the physiotherapist's examination by amplifying diagnostic precision and supporting differential decision-making. Providing timely, appropriate, and accurate diagnostic information enhances patient care by guiding appropriate intervention selection and facilitating targeted referral when indicated (10, 13, 43, 45, 46, 48).

EDX testing aligns naturally with physiotherapists' expertise in evaluating movement-related dysfunctions and the associated pathophysiologic changes in the neuromuscular system (Tables 1, 2) (8, 10, 13, 16–18, 47, 49). Consistent with World Physiotherapy's policy statements, the performance and interpretation of EDX testing by physiotherapists fall within the professional scope in jurisdictions where regulations and educational standards authorize such practice (35).

**Table 1:** Diagnostic role of electrodiagnostic (EDX) testing and ultrasound imaging (USI) in the evaluation of conditions commonly encountered in orthopedic/sports practices.

Conditions	EDX testing	USI
ORTHOPEDIC/SPORTS		
Muscle Strain (e.g., acute, myositis ossificans)	N/A^c^	Diagnostic^a^
Muscle Atrophy (e.g., disuse, age-related sarcopenia)	Adjunctive^b^	Diagnostic
Myopathy (e.g., dermatomyositis, polymyositis)	Diagnostic	Diagnostic
Ligament Sprain (e.g., collaterals in elbow and knee, ulnar collateral in thumb)	N/A	Diagnostic
Tendinitis/Tendinopathy (e.g., acute, degenerative, calcific)	N/A	Diagnostic
Tendon Tear (e.g., rotator cuff, biceps, Achilles, gluteal)	N/A	Diagnostic
Bursitis (e.g., subacromial, greater trochanteric, acute, calcific)	N/A	Diagnostic
Edema/Effusion (e.g., soft tissue and joint injuries)	N/A	Diagnostic
Bleeding Conditions (e.g., contusions, hematomas, hemarthrosis)	N/A	Diagnostic
Soft Tissue Masses (e.g., ossificans, ganglion cysts, lipomas, hemangiomas)	N/A	Adjunctive
Osteoarthritis (e.g., thumb carpometacarpal, osteophytes, enthesophytes)	N/A	Diagnostic
Rheumatoid Arthritis (e.g., joint erosions, synovitis, synovial thickening)	N/A	Diagnostic
Stress Fracture (e.g., medial tibial stress, metatarsals)	N/A	Diagnostic
Fracture (e.g., ribs, metacarpals, metatarsals)	N/A	Diagnostic
Pain Conditions (e.g., cervicogenic headache, CRPS, fibromyalgia)	Adjunctive	N/A

CRPS, complex regional pain syndrome.

a Diagnostic: considered among primary tests routinely used to rule-in or rule-out presence of condition.

b Adjunctive: considered among secondary tests routinely used to assist in differential diagnosis of condition.

c N/A: not considered among tests routinely used for diagnosis or differential diagnosis of condition.

**Table 2:** Diagnostic role of electrodiagnostic (EDX) testing and ultrasound imaging (USI) in the evaluation of conditions commonly encountered in neurologic practices.

Conditions	EDX testing	USI
NEUROLOGIC		
Entrapment Neuropathy (e.g., carpal tunnel, cubital tunnel, tarsal tunnel)	Diagnostic^a^	Adjunctive^b^
Radiculopathy (i.e., cervical and lumbosacral)	Diagnostic	N/A^c^
Polyneuropathy (e.g., alcoholic, CIDP, diabetic, genetic)	Diagnostic	Adjunctive
Plexopathy (i.e., brachial and lumbosacral)	Diagnostic	Adjunctive
Neuromuscular Junction Disorder (e.g., botulism, LEMS, myasthenia gravis)	Diagnostic	N/A
Motor Neuron Disease (e.g., ALS, spinal muscular atrophy, polio)	Diagnostic	Adjunctive
Nerve Subluxation/Dislocation (e.g., ulnar nerve at elbow)	N/A	Diagnostic
Nerve Sheath Tumors (e.g., Schwannomas, neurofibromas)	Adjunctive	Diagnostic
Neuroma (e.g., traumatic, benign, genetic)	Adjunctive	Diagnostic
Upper Motor Neuron Conditions (e.g., cerebral vascular accident, SCI)	Adjunctive	N/A

CIDP, chronic inflammatory demyelinating polyneuropathy; LEMS, Lambert-Eaton myasthenic syndrome; ALS, amyotrophic lateral sclerosis; SCI, spinal cord injury.

a Diagnostic: considered among primary tests routinely used to rule-in or rule-out presence of condition.

b Adjunctive: considered among secondary tests routinely used to assist in differential diagnosis of condition.

c N/A: not considered among tests routinely used for diagnosis or differential diagnosis of condition.

On March 1, 1953, *The Physical Therapy Review* published a manuscript entitled “Electromyography in the Practice of Physical Medicine, The Role of the Physical Therapist”, an article that followed a national presentation on the same topic the preceding June at the APTA's annual conference in Philadelphia (49). This historic publication marked the first documented case of a physiotherapist performing needle electromyography, underscoring its clinical value in hospital-based care. By the mid-1970s physiotherapists routinely performed EDX testing in both military and civilian healthcare systems under established credentialing frameworks and in collaboration with referring physicians.

In 1982, the APTA House of Delegates approved board certification in Clinical Electrophysiology, formally recognizing and credentialing EDX testing by physiotherapists. In 1986 the American Board of Physical Therapy Specialties administered the first Clinical Electrophysiology Specialist Certification Examination. Currently, 46 U.S. states either explicitly permit, remain silent on, or have attorney general opinions affirming EDX testing as within physiotherapy scope, with only Hawaii, Michigan, Nebraska, and New Jersey maintaining prohibitions (44).

### 2.2 Ultrasound imaging

Point-of-care USI is a safe, cost-effective, and portable diagnostic tool used to evaluate the integrity of the neuromusculoskeletal system (43, 45, 46, 50–52). Conducted as an extension of the clinical examination and not as a laboratory procedure, USI is a dynamic imaging modality capable of evaluating soft tissue and joint structure, morphology, and movement of muscular, skeletal, neural, vascular, and articular tissues in *real-time* including during active or passive movements and during “sonopalpation” (12, 14, 52). Compared to other static imaging techniques, USI has particular diagnostic value for evaluating peripheral nerves, tendons and tendon insertions, contusions, hematomas, myositis ossificans traumatica, rib fractures, stress and avulsion fractures, and joint erosions and intra-articular osteochondral irregularities associated with rheumatoid and osteoarthritis and hemophilic arthropathies (7, 9, 11, 12, 52–56). Moreover, the dynamic nature of USI enables detection and differentiation of joint effusion and hemarthrosis—an advantage over magnetic resonance imaging, which cannot distinguish these fluid types using T2-weighted sequences (9, 12, 52).

The four broad categories of USI applications in physiotherapy practice include diagnostic, interventional, rehabilitative, and research (50, 51), each employing B-mode (“brightness”) or grayscale and Doppler mode (i.e., color or power) technologies to produce images that provide clinicians with structural and morphological information about a variety of soft tissues and fluid in the neuromusculoskeletal, cardiovascular, and cardiopulmonary systems, spanning applications from pelvic health to conditions involving edema, effusion, inflammation, and neovascularization (9, 52, 57–59).

USI has rapidly gained popularity among providers in orthopedic/sports and neurologic settings, and is now recognized as a reliable and valid diagnostic tool that enhances diagnostic precision, informs intervention planning, improves the safety and effectiveness of image-guided procedures, and enables longitudinal tracking of soft tissue change (Tables 1, 2) (15, 43, 60, 61). Additionally, USI complements EDX testing by providing correlative information on peripheral nerve morphology, echotexture, and mobility abnormalities (e.g., increased cross-sectional area, edema, perineural thickening, and neovascularity) (6, 7, 11, 43, 52, 53, 62–64). These clinical integrations reinforce World Physiotherapy's vision for autonomous diagnostic reasoning supported by evidence-based technologies in education and practice (35).

Karl Dussik's first musculoskeletal ultrasound report in 1958, through physiotherapists' initial use in 1980, to formal recognition by the American Institute of Ultrasound in Medicine in 2017, charts a remarkable evolution of the profession's engagement with diagnostic imaging (51, 65, 66). Globally, physiotherapists are increasingly recognized as qualified users of point-of-care USI when supported by appropriate education and training. This trajectory mirrors World Physiotherapy's assertion that advanced practice roles improving diagnostic access and accuracy fall within the evolving global scope of the profession.

Inteleos™, the gold-standard credentialing body for medical imaging certifications and parent organization to the Alliance for Physician Certification & Advancement™ (APCA) and the Point-of-Care Ultrasound (POCUS) Certification Academy™, recognizes physiotherapists as advanced care providers eligible for their certifications. Notably, physiotherapists are eligible and encouraged to pursue the RMSK® credential (Registered in Musculoskeletal® sonography), the physician-level certification in diagnostic musculoskeletal sonography offered through APCA. Additionally, physiotherapists may also obtain certifications through the POCUS Certification Academy across multiple specialties including cardiac, vascular, pulmonary, visceral, pelvic, abdominal, and musculoskeletal USI (Explore POCUS Certifications, http://www.pocus.org).

## 3 Instrumented diagnostics in primary care physical therapist practice: the future

### 3.1 Improved diagnostic precision

To accomplish the vision of the physiotherapy profession and gain widespread acceptance as first-contact, primary care providers, the skilled use and interpretation of advanced point-of-care diagnostic tests that extend the clinical examination is necessary because many clinically-based tests and measures lack sufficient diagnostic value to produce meaningful shifts in post-test probabilities (4, 5).

As advancements in healthcare technology continue to provide sophisticated tools for patient care, it is imperative that the physiotherapy profession evolves in parallel by modernizing clinical practices to integrate advanced diagnostic and evaluative methods which enhance the precision, efficacy, and relevance of physiotherapy care. By embracing innovative technologies, first-contact physiotherapists can elevate standards of care, align with modern healthcare demands, and optimize patient outcomes. Such innovation aligns with World Physiotherapy's emphasis on leveraging emerging diagnostic tools to strengthen clinical reasoning, promote autonomy, and meet population health needs across diverse healthcare systems.

**Figure 1:**
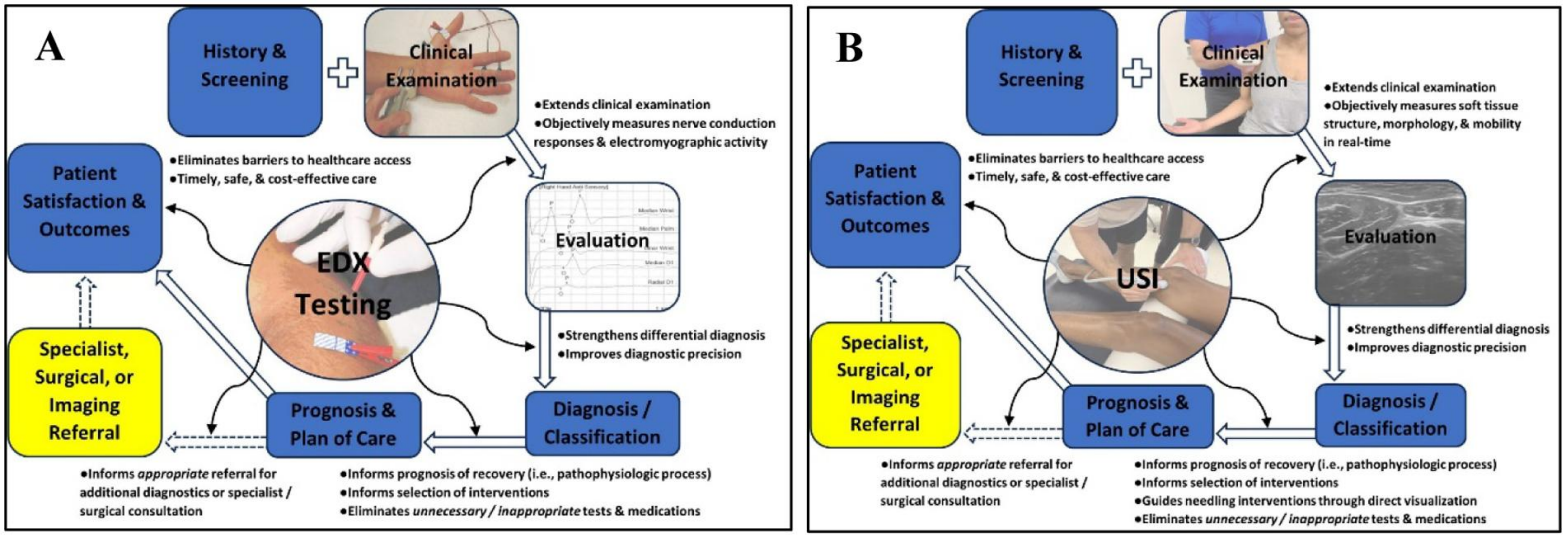
Role and value of point-of-care electrodiagnostic (EDX) testing **(A)** and ultrasound imaging **(B)** in the medical management of patients with neuromusculoskeletal-region complaints and dysfunction.

The findings from EDX testing (Figure 1A) and USI (Figure 1B) examinations enhance diagnostic precision, strengthen the differential diagnostic process, inform selection of appropriate interventions, and improve overall medical management through timely and appropriate referral to outside providers for consultation, co-management, or for additional diagnostic testing or surgery (6, 7, 17, 18, 43, 67–69). Clinicians regularly encounter uncertainty when examining patients with neuromusculoskeletal-related pain and dysfunction, a problem particularly relevant in orthopedic/sports and neurologic practices that employ the highest density of physiotherapists, with these three specialty areas representing nearly 80% of all board-certified clinical specialists in the U.S. Referred and radiating symptoms and non-neuromusculoskeletal pathologies can mimic a patient's regional signs and symptoms and pose diagnostic challenges for clinicians seeking to identify the true cause and location of a patient's underlying condition (4, 5, 43, 45, 46).

Clinical frameworks in musculoskeletal care emphasize the importance of an initial screening examination followed by targeted testing to clarify the source of symptoms. This staged approach recognizes that multiple pathologies may produce overlapping symptom patterns within a given region (70). For example, in the upper extremity, paresthesia in the medial hand may be caused by ulnar neuropathy at the wrist or elbow, lower cervical radiculopathy, inferior trunk or medial cord brachial plexopathy (e.g., neurogenic thoracic outlet syndrome), or non-neuromusculoskeletal conditions such as a space occupying mass (e.g., Pancoast tumor) or peripheral arterial disease. Similarly, in the lower extremity, pain in the buttock region may be caused by lumbar facet or disc dysfunction or injury, sciatic neuritis or neuropathy, hip or gluteal muscle dysfunction or injury, or non-neuromusculoskeletal conditions such as a space occupying mass or an organ dysfunction or injury (4, 5, 43, 45–47).

Consistent with this staged approach, the addition of EDX testing and USI clarifies and extends the clinical examination by accurately identifying and localizing the underlying pathophysiologic process related to a patient's symptomatic presentation and movement dysfunction. Utilizing instrumented diagnostic testing in contemporary physiotherapist practice provides a more comprehensive approach to the examination and evaluation of patients and can better inform diagnosis, prognosis, and intervention selection, and guide medical management including referral when necessary (17, 43, 45, 46). These earlier examples, like distinguishing ulnar neuropathy from cervical radiculopathy or identifying vascular vs. neuromuscular causes of lower extremity symptoms, illustrate the clinical value of EDX testing and USI in differentiating overlapping symptom presentations.

As the physiotherapy profession remains focused on establishing a larger role as first-contact, primary care providers, it is vital that the opportunities for education and training align with those of other advanced care practitioners, including the language that physiotherapists use when communicating with other healthcare professionals. Thought leaders and professional bodies have emphasized the need for physiotherapy-specific diagnostic labels that are accurate, descriptive, and actionable, particularly when considering the profession's expanding role into first-contact, primary care practice (3). The importance of accurate and descriptive diagnostic labels comes into clearer focus when considering the integration of instrumented point-of-care diagnostic testing in contemporary physiotherapist practice, where innovative technologies assist clinicians in defining and refining physiotherapy-related diagnostic categories.

The process of defining and refining diagnostic labels requires data in the form of normative values or cut-point criteria, standardized testing protocols, and community guidelines that improve test reliability, validity, and clinical utility across examiners and facilities for a variety of patients. This process requires robust analytic methods and quantitative image analysis (e.g., radiomics for imaging-related tests) and deliberate integration of EDX- and USI-based evidence into the development of clinical prediction rules, clinical practice guidelines, and economic analyses of physiotherapy services (71, 72).

### 3.2 Guiding interventions and informing prognosis

In addition to enhancing diagnostic precision, both EDX testing and USI play pivotal roles in guiding the selection of appropriate interventions and informing prognosis. These instrumented point-of-care diagnostic tests provide clinicians with objective, reproducible data that refine the selection and dosing of interventions.

For example, USI can guide the application of neuromuscular electrical stimulation by helping clinicians titrate stimulation intensity based on *real-time* visualization of muscle activation, thereby improving treatment precision and efficacy—an application particularly useful in patients with multifidus dysfunction associated with chronic low back pain (73).

Likewise, EDX testing and USI findings can influence clinical decisions related to exercise dosing, manual therapy parameters, and physical activity progression, especially in patient populations with bleeding disorders like hemophilia where careful loading is paramount. USI provides direct visualization of joint effusion, synovial hypertrophy, and muscle hematomas, enabling safer and more individualized clinical decision-making (9).

USI also provides prognostic value, as it can quantify tissue morphology and healing over time. In orthopedic settings, measurements such as tendon gap distance and tendon length following rupture (e.g., Achilles tendon rupture) can help predict functional recovery timelines and the likelihood of conservative vs. surgical outcomes. Serial USI can track tendon regeneration, fibrosis, or persistent gapping, aiding prognostic stratification and shared decision-making (74).

Similarly, EDX findings, including evidence of active denervation, reinnervation patterns, or chronic neurogenic changes—provide insight into the chronicity of nerve injury and the underlying healing mechanisms (e.g., axonal sprouting vs. direct reinnervation), thereby informing both prognosis and rehabilitation planning (43, 45, 46).

### 3.3 Enhanced entry-level and post-graduate training

Contemporary physiotherapist practice requires expanded use of EDX testing and USI, a goal that will be achieved by increasing entry-level and post-graduate education in these instrumented point-of-care diagnostic tests—ideally developed specifically for physiotherapists rather than adapted from physician-oriented materials (58, 59, 75–79).

Expanded opportunities for physiotherapists to learn the theoretical and practical applications of EDX testing and USI—two of the few instrumented diagnostic tests currently available that physiotherapists can perform, interpret, and receive insurance reimbursement for in many regions–will drive greater clinical adoption and further strengthen the evidence-based rationale supporting modern physiotherapist practice. First-contact, primary care physiotherapist practice is safe and effective (21, 22, 24, 25, 37). Importantly, there are no known records of an increase in adverse events associated with physiotherapists performing EDX testing or USI—the latter an inherently safe imaging modality with no ionizing radiation and minimal thermal or mechanical side effects delivering a fraction of the energy output of therapeutic ultrasound (10, 12–14, 44, 50, 52, 57, 61).

While many physiotherapy education programs currently introduce students to EDX testing, USI, and other imaging or laboratory-based methods, this exposure rarely culminates in clinical proficiency at the time of graduation. Students may observe or engage in isolated skill practice, but few develop the diagnostic reasoning or technical competence necessary to apply these valuable point-of-care diagnostic tools independently in practice. Integration of EDX testing and USI should include a deliberate scaffolding of theoretical knowledge, hands-on psychomotor skill development, and progressive competency-based assessments that lead to measurable proficiency. Just as examination and manual therapy skills are developed across a sequence of courses and clinical experiences, the same educational model should be considered for point-of-care diagnostic testing.

This approach aligns with competency-based education principles and reflects global expectations, such as those articulated in World Physiotherapy's *Education Framework*, which calls for physiotherapists to be prepared as autonomous, first-contact practitioners capable of using a range of diagnostic tools to support safe and effective patient management (80). Building true proficiency rather than introductory exposure will better equip graduates to contribute the profession's modernization through improved diagnostic accuracy, cost containment, and interprofessional collaboration.

Today, more medical schools are integrating USI into their program curricula, a trend mirrored by entry-level physiotherapy education in the U.S (51, 75, 78). Inclusion of lecture- and laboratory-based instruction focused on the application, performance, and interpretation of EDX testing and USI is feasible and can often be accomplished with minimal curricular restructuring by embedding content within existing courses across the program sequence (68, 75).

Such curricular integration is supported by World Physiotherapy's *Education Framework*, which emphasizes that entry-level physiotherapy education should prepare graduates to function autonomously, using diagnostic tools within their scope to enhance patient care and collaboration (80). This approach aligns with several standards outlined by the *Commission on Accreditation in Physical Therapy Education* (CAPTE), including 7A (diagnostic imaging), 7D16 (screening for referral), 7D19 (tests/measures), 7D22 (diagnosis for medical management), 7D35 (direct access), and 7D39 (interprofessional collaborative practice) (81).

Despite growing interest in integrating EDX testing and USI into entry-level physiotherapy education, significant barriers remain, particularly in accelerated programs with compressed timelines, limited lab hours, and high student-to-faculty ratios. Embedding meaningful instruction in these areas—which requires supervised skill acquisition—demands innovative curricular design and underscores the need for post-graduate training pathways. Addressing these challenges requires collaboration with key professional bodies to shape curricular standards, accreditation expectations, and licensure examinations.

Current criteria include language on diagnostic imaging, screening for referral, tests and measures, and interprofessional collaboration, but lack specificity regarding the depth, breadth, and benchmarks of competency in EDX testing and USI performance and interpretation.

Importantly, it is unclear whether licensure examinations worldwide evaluate competency in EDX testing or USI in any substantive way. This gap necessitates periodic practice analyses to ensure alignment between licensure content and real-world practice demands. However, there is no current evidence that these competencies are systematically assessed in recent examination cycles. The disconnect between clinical utility and evaluative benchmarks create a structural barrier to curriculum development, particularly in programs designed around licensure preparation.

To bridge these gaps, the physiotherapy profession must advocate for clearer national curricular benchmarks, competency-based performance standards, and licensure content that reflect the evolving scope of practice. Without such alignment, well-intentioned faculty may struggle to justify adding contemporary content–including point-of-care diagnostic testing–that lacks external validation or assessment.

While many entry-level programs lack board-certified specialists in EDX testing and USI—a limitation that constrains teaching capacity—modern education models using online and hybrid formats enable synchronous and asynchronous learning and remote feedback for hands-on skill acquisition. Embedding EDX testing and USI content longitudinally across courses such as gross anatomy, surface palpation, diagnostic imaging, and musculoskeletal and neurologic physiotherapy allows students to accumulate meaningful hours of training across a typical 24- or 36-month program.

Alternatively, programs may provide baseline theoretical and practical knowledge to all students, followed by elective courses for those seeking advanced hands-on experience. Ideally, students would have opportunities for clinical placements with instructors experienced in EDX testing and/or USI to gain deeper understanding of how these diagnostic tools extend the physiotherapy examination and evaluation.

Following graduation, early- and mid-career physiotherapists can pursue additional training through *American Board of Physical Therapy Residency & Fellowship Education* (ABPTRFE)-accredited programs in Clinical Electrophysiology (Online Directory of Programs, accreditation.abptrfe.org). There is currently one doctoral-level program in clinical electrophysiology available in the U.S. Although few formal residency pathways exist in USI, several advanced fellowship, diploma, and certificate options are available. Additionally, ABPTRFE-accredited residencies in Orthopaedics, Sports, and Women's Health and fellowships in Spine, Sports Division, and Upper Extremity Athlete specialties often include USI learning experiences based on their patient populations and commitment to contemporary practice.

Practicing clinicians seeking to incorporate EDX testing and USI can attend a range of in-person, online, and hybrid continuing education courses(1.5-7 days; 12-56 h) (77). Mentorship programs also exist for clinicians and educators interested in advancing their knowledge and clinical integration of these technologies.

Although formal data on EDX testing and USI prevalence remain limited, existing studies identify consistent barriers: lack of structured training, high equipment costs, inconsistent reimbursement, limited institutional support, and scope-of-practice ambiguity—particularly in regions with variable regulation of diagnostic testing by non-physician providers.

Ellis et al. (2020), in an international survey of physiotherapists from 49 countries, found that 38% reported using USI in some capacity (e.g., biofeedback, diagnostic, research, teaching) but cited confusion regarding scope of practice and lack of training as primary barriers (82). Similarly, Savage et al. (2024) reported that lack of qualified faculty, limited curricular space, and equipment costs were the main obstacles to inclusion in U.S. DPT programs (75).

Furthermore, regulatory and reimbursement inconsistencies likely influence both EDX testing and USI utilization. Although comprehensive utilization data are lacking, existing publications suggest that structural and financial barriers–not lack of clinical interest or professional readiness–remain the chief obstacles to broader implementation of point-of-care diagnostic tools by physiotherapists.

### 3.4 Advocacy and payment

Broader implementation of EDX testing and USI in contemporary physiotherapist practice is inseparable from ongoing advocacy efforts and strategic reform of reimbursement models. Globally, the degree to which physiotherapists are recognized as autonomous providers capable of ordering or performing diagnostic testing varies by jurisdiction and is often limited by outdated regulations or insufficient recognition among payers and policymakers.

Advocacy initiatives at the national and international levels are needed to expand physiotherapists' diagnostic authority, provide regulatory clarity, and establish payment parity with other advanced practice providers. World Physiotherapy and several national professional bodies have called for policy frameworks formally acknowledge recognize the diagnostic authority of physiotherapists—particularly within models of care emphasizing first-contact, cost efficiency, and workforce flexibility.

Reimbursement for EDX testing and USI also presents a barrier to clinical adoption in some regions and settings. While some countries already allow billing for these services when performed by appropriately credentialed physiotherapists, many do not. Without consistent funding mechanisms or recognition by third-party payers, adoption of these valuable point-of-care diagnostic tools will remain limited, regardless of their clinical utility or the profession's readiness to modernize.

Future progress will depend not only on educational expansion but also on coordinated interprofessional collaboration, health economic research, and sustained policy advocacy demonstrating that physiotherapist-administered point-of-care diagnostic testing is safe, appropriate, effective, and adds measurable value to healthcare systems worldwide. Advancing these aims will require alignment among educational institutions, clinicians, professional associations, and policymakers.

### 3.5 Cost-effectiveness

The expanded use of EDX testing and USI as instrumented point-of-care diagnostic tools is a natural fit that builds upon the excellent examination and evaluation skills of physiotherapists. Increasing the use of these innovative technologies will not only modernize the physiotherapy profession but also elevate the standing of contemporary physiotherapist practice as an indispensable interdisciplinary asset within the larger medical community.

Increasing access to appropriate, timely, and cost-effective diagnostic testing services helps remove key barriers faced by patients in increasingly strained healthcare systems worldwide (21, 23, 31). Patients with neuromusculoskeletal-related complaints often experience frustration when navigating a complex healthcare landscape—particularly those in rural and medically underserved areas who face difficulty scheduling primary and specialty care appointments and have limited geographic access to diagnostic and interventional services—factors that result in care delays lasting weeks or even months (21, 23, 31).

Compared to first-contact physiotherapist care, traditional medical management of neuromusculoskeletal-related complaints is typically less efficient, more time-consuming, and more expensive, leading to higher rates of unnecessary medications, diagnostic testing, and surgeries. Traditional care often involves multiple referrals, repeated clinic visits, and redundant testing—factors that can confuse patients, increase costs, and contribute to suboptimal outcomes (19, 21–23, 25–28, 32, 34, 37, 39). In contrast, direct-access physiotherapy services provide timely, cost-effective, and high-quality care for patients with neuromusculoskeletal-related complaints, significantly reducing the need for outside referrals and redundant visits (21, 23, 24, 26, 32–34).

According to the Association of American Medical Colleges, the current supply-demand imbalance could lead to a shortage of more than 40,000 primary care physicians by 2036—and as many as 86,000 physicians overall—in the U.S. In addition to provider shortages, wait times for primary care providers visits increased by 30% from 2014 to 2017. (30) As a result, patients increasingly seek care elsewhere, contributing to a 30% decline in per capita primary care visits from 2002 to 2015 and a corresponding rise in visits to urgent care centers, retail clinics, and emergency departments (23). Moreover, it is estimated that primary care providers would need 22 h per day to deliver optimal care—an untenable figure given burnout rates exceeding 50% among family medicine physicians. Despite these challenges, direct access to physiotherapist services offers a promising solution: patients experience better outcomes at lower costs, higher satisfaction, reduced imaging and medication use, and no increase in adverse events. Direct access also reduces physician workload by managing a substantial portion of primary care demand, notable given that patients with neuromusculoskeletal complaints represent approximately 27% of all primary care visits (23).

Point-of-care diagnostic testing services also provide enhanced opportunities for patient education. Clear and timely communication from providers helps patients understand their condition and prescribed interventions, improving compliance and outcomes—including cases that warrant referral to an outside provider. For some patients, serial testing enables clinicians to track tissue changes related to healing, regeneration, or degeneration, offering objective measures of progress that can be interpreted alongside clinical examination findings, functional performance, and patient-reported outcomes measures (4, 5, 43, 45, 46, 52).

## 4 Conclusions

Innovative healthcare technologies provide sophisticated tools for patient care, and it is imperative that the physiotherapy profession evolves to meet the emerging needs of contemporary clinical practice. Modernizing the profession through the integration of advanced diagnostic and evaluative tools is essential to enhance the precision, efficacy, and relevance of physiotherapy as a first-contact, primary care profession. Importantly, we advocate not for the indiscriminate use of instrumented diagnostic testing, but for the responsible integration of these powerful point-of-care tools into clinical practice through competency-based education, evidence-informed clinical decision-making, and appropriate scope of practice guidelines. These technologies are most effective when used to resolve diagnostic uncertainty, and where diagnostic clarity has the potential to meaningfully influence treatment planning, referral decisions, or prognosis. The integration of point-of-care diagnostic testing should always reflect a commitment to evidence-based, patient-centered care. We emphasize that the integration of EDX testing and USI in contemporary physiotherapy practice is not intended to increase the *volume* of diagnostic testing, but to improve its *appropriateness and precision* when uncertainty persists. Properly applied, these valuable point-of-care diagnostic tools can reduce the reliance on higher cost imaging modalities and contribute to more efficient patient-centered care—particularly for patients in rural and medically underserved areas where physiotherapists may represent the only accessible medical provider of such services.

The physiotherapy profession must continue to elevate its standards to meet global healthcare demands and ensure optimal patient outcomes in an ever-changing clinical landscape. EDX testing and USI are instrumented point-of-care diagnostic tools with established pathways for training and credentialing. Contemporary physiotherapy practice requires their integration through expanded education and training in entry-level programs and robust post-graduate opportunities. Overcoming barriers such as training access, equipment cost, and reimbursement variability will be critical for achieving equitable and evidence-informed implementation of EDX testing and USI within physiotherapy practice.

Growing the use of EDX testing and USI by physiotherapists in clinical practice, particularly in the clinically dense orthopedics/sports and neurology specialties, will further establish physiotherapy as a first-contact, primary care profession and elevate their global standing as indispensable interdisciplinary providers within the larger international medical community. Positioning physiotherapists to lead in models of care that include point-of-care diagnostics reflects global policy efforts to improve access, promote timely interventions, and reduce system inefficiencies. Physiotherapy as a first-contact, primary care profession can better meet the emerging needs of patients and society in increasingly strained healthcare systems by providing timely, appropriate, and cost-effective point-of-care diagnostic and interventional services—and by removing barriers to quality, affordable care driven by primary and specialty care provider shortages.

## Data Availability

The original contributions presented in the study are included in the article/Supplementary Material, further inquiries can be directed to the corresponding author.
